# Lab-scale photobioreactor systems: principles, applications, and scalability

**DOI:** 10.1007/s00449-022-02711-1

**Published:** 2022-03-18

**Authors:** Philipp Benner, Lisa Meier, Annika Pfeffer, Konstantin Krüger, José Enrique Oropeza Vargas, Dirk Weuster-Botz

**Affiliations:** 1 Department of Energy and Process Engineering, Chair of Biochemical Engineering, Technical University of Munich, TUM School of Engineering and Design, Boltzmannstraße 15, 85748 Garching, Germany; 2 Technical University of Munich, TUM School of Engineering and Design, Boltzmannstraße 15, 85748 Garching, Germany; 3 Technical University of Munich, TUM-AlgaeTec Center, 85521 Taufkirchen, Germany

**Keywords:** Photobioreactor, Microalgae, Lab-scale reactors, Scale-up

## Abstract

Phototrophic microorganisms that convert carbon dioxide are being explored for their capacity to solve different environmental issues and produce bioactive compounds for human therapeutics and as food additives. Full-scale phototrophic cultivation of microalgae and cyanobacteria can be done in open ponds or closed photobioreactor systems, which have a broad range of volumes. This review focuses on laboratory-scale photobioreactors and their different designs. Illuminated microtiter plates and microfluidic devices offer an option for automated high-throughput studies with microalgae. Illuminated shake flasks are used for simple uncontrolled batch studies. The application of illuminated bubble column reactors strongly emphasizes homogenous gas distribution, while illuminated flat plate bioreactors offer high and uniform light input. Illuminated stirred-tank bioreactors facilitate the application of very well-defined reaction conditions. Closed tubular photobioreactors as well as open photobioreactors like small-scale raceway ponds and thin-layer cascades are applied as scale-down models of the respective large-scale bioreactors. A few other less common designs such as illuminated plastic bags or aquarium tanks are also used mainly because of their relatively low cost, but up-scaling of these designs is challenging with additional light-driven issues. Finally, this review covers recommendations on the criteria for photobioreactor selection and operation while up-scaling of phototrophic bioprocesses with microalgae or cyanobacteria.

## Introduction

After the earlier ‘green trend’ in the 1980s, bio-based chemicals, such as oleochemicals, gained increasing attention as a sustainable alternative to fossil fuel-based products [1]. The EU Commission identified the chemical industry as one of the closest industries to the EU Green Deal, due to its impact on the end-use sector. The Commission intends to present a chemical strategy for sustainability to protect citizens and the environment from hazardous chemicals, as well as to promote innovation for the development of safe and sustainable alternatives. The priority is to develop new technologies without directly or indirectly using valuable land for plants, food, or fossil fuels [2]. Biotechnological methods can pave the way to more sustainable chemical products of renewable resources [3]. Photosynthetic microorganisms such as microalgae and cyanobacteria have become subjects of interest recently. Converting carbon dioxide provides an environmental advantage over heterotrophic organisms [4], as CO_2_ sequestration reduces the increase of global warming [5]. Phototrophic microorganisms may become cell factories for the biological synthesis of bioactive compounds. In a study, supported by the European Union, 33 microalgae strains were analyzed as new potential production hosts for active compounds for human therapeutics [6]. Some species of microalgae, particularly brown algae, have also been in the spotlight of food production, thanks to their nutritional value. These organisms contain complex polysaccharides, minerals, proteins, and vitamins as well as diverse phytochemicals, which have interesting therapeutic properties [7]. Bioactives with antioxidative, anti-inflammatory, immunomodulatory, antihypertensive, anticancer, and anticoagulant effects were found in red algae and *Spirulina* [8–10]. With the increasing demand for environmentally friendly alternatives, the development of biofuel and biolubricants has become a common research topic. Lipids produced from microalgae or other microorganisms are the basis for this production [11–13]. While plants like rapeseed are nowadays used for lipid production, microalgae seem to be a promising alternative as they do not compete with the food and grow fast [14, 15]. In wastewater treatment applications, mixotrophic microalgae can also be implemented as they use different organic and inorganic substances that would be considered a contaminant otherwise, for their growth [16]. The application of mixotrophic microalgae in combination with wastewater treatment are reviewed in [19]. Aquatic plants and microalgae are also used for phytoremediation, absorbing pollutants such as nitrogen and phosphorus, degrading organic matter, and accumulate heavy metals in their biomass [17, 18].

On an industrial scale, microalgae are usually cultivated in open-pond systems due to the low operational cost; however, the ponds offer an insufficient control of reaction conditions as well as possible contamination from harmful microorganisms [19]. In comparison, closed photobioreactor systems usually offer a higher yield of biomass and product as well as higher photosynthetic efficiency and lower water loss thanks to the controlled environment, but are much more expensive [20].

Ideally, photobioreactors would have a perfect mixing of substrates with little hydrodynamic shear stress for the cells, no dead volume, sufficient gas–liquid mass transfer for CO_2_ absorption and O_2_ release, and, in the case of photobioreactors, each microalgae cell would have access to optimal light absorption in any position inside the bioreactor [21]. However, in reality, ideal photobioreactor conditions are impossible to achieve, so different designs of closed photobioreactors are available, each with different techniques to come as close as possible to these ideal conditions to ensure optimal growth and production during the process [19]. In this review, the focus will be on laboratory-scale photobioreactors, which range in volume from a few liters [22] to microliters [23] and are characterized by their use in research instead of the production of consumer goods.

One of the crucial requirements for a photobioreactor is to provide enough light to allow for the growth of the microalgae or cyanobacteria culture. Low light intensities do not deliver enough energy for optimal growth and high light intensities lead to photoinhibition of the algae light-harvesting system [24, 25]. The quantity of light that is emitted by the light source is not the same as what gets absorbed by the photosystems of the cells. Thus, there are different state variables to measure light quantity in the photobioreactor. One is the light intensity. It represents the luminous flux in µmol m^−2^ s^−1^ that is irradiated from the light source to the surface of the reactor. There, on the surface, it is also referred to as incident photon flux density. Incident light intensity is easy to measure, can be compared between all different kinds of reactors, and is a basic light parameter mentioned in almost all the literature. Unfortunately, the informative value of the incident photon flux density is limited because light gets attenuated over distance in the suspension as the growing cells absorb the light and shade each other in the culture. Light attenuation should be taken into account. For example, Pfaffinger et al. rely on the mean light intensity that can be calculated depending on the layer thickness of the suspension and biomass concentration of the culture [26].

While on an industrial scale the sun often illuminates the photobioreactors, lab-scale photobioreactors are illuminated by artificial light. It is possible to reach higher biomass or product concentration with the light of a certain wavelength: white, red, and blue light have shown to be sufficient for laboratory-scale cultivation [27, 28]. But because industrial-scale cultivation usually takes place under light-limited conditions, which allow for good process control, growth behavior under a light limitation is also investigated in laboratory photobioreactors [29, 30]. Additional fixtures, that channel the light inside the photobioreactor can enhance light distribution and are easy to scale up by just increasing their number for higher volumes [31, 32]. An alternative to engineering the illumination efficiency of the photobioreactor is to engineer the light-harvesting system of the cultivated microorganism [33]. This can be via genetic modification by reducing chlorophyll b synthesis or red-shifting of the light-harvesting system, for example [34, 35]. Those methods are quite new and still under development, but they have already led to promising results regarding enhanced algae growth under high light intensity or the light of a certain wavelength [36–38].

Scaling-up photobioreactor processes present higher complexity compared to conventional reactors, as the heterogeneity of light intensity and availability is a critical factor [39]. Transfer from laboratory to large-scale cultivation of microalgae processes requires careful planning [40, 41]. Besides the crucial light supply, the optimization of state variables such as pH, temperature, CO_2_ supply and O_2_ release, inoculum concentration in batch processes and nutrient composition is also required before any scale-up can be considered [39, 42]. Before cultivating at the production scale, the cultivation method needs to be chosen wisely by weighing the different cultivation systems against each other [40]. Scaling factors must be considered from the beginning of the production system and the equipment must be designed accordingly [41]. Lab-scale experiments considering these state variables are imperative to pursue a successful scale-up [39–42].

Morschett et al. already published a mini review on micro photobioreactors that focuses mainly on microtiter plates, while other reactor types are marginally covered [43]. This review’s claim is to integrate the new insights on microtiter plate cultivation of photosynthetic microorganisms since then and combine them with more detailed information on other laboratory size photobioreactors, to evaluate their advantages and disadvantages in scientific application. Beyond that, this review highlights the potentials of scale-up microalgae cultivation processes based on data generated with the laboratory photobioreactors.

Regarding the scale-up and its concept to a successful industrial application similar review papers also exist [29, 44, 45]. Pruvost et al. discuss parameters to consider when designing and operating microalgal cultivation systems and how appropriate engineering rules can support optimal system design and operation. Moreover, in their review, the focus lies on explaining the influencing parameters to better understand the interrelationship. In previous reviews, the focus is often on the various types of reactors used in industry, their advantages, and disadvantages, and how to handle them. It is often emphasized that the technology transfer from small scale to large scale is essential, but not how and according to which rules this is best approached [39, 46]. On the other hand, most published studies regarding photobioreactors at the laboratory scale only have a very restricted theoretical outlook on scaling up [22, 29, 47]. The technology for the large-scale cultivation of phototrophic microorganisms already exists. Being feasible on this scale is more critical and depends largely on the correct choice of the state variables and the right technology transfer from the laboratory to the industrial scale [48, 49]. The different lab-scale photobioreactors can simulate large-scale conditions in different ways, which is the reason why numerous different reactors exist, all with their special advantages and disadvantages. This review intends to summarize which type of laboratory-scale photobioreactor can simulate large-scale conditions, to what degree, and to give recommendations about which reactor might be used for pre-scale-up experiments. The section on scale-up is intended to provide guidelines based on the existing problems that should be considered during a scale-up.

## Lab-scale photobioreactor systems

### Microfluidic photobioreactors

A recent option for studying microalgae are illuminated microfluidic photobioreactors. In these chips, several culture compartments are connected with a fluidic channel, to allow the run-in of microalgae as well as nutrients. Usually built with polydimethylsiloxane (PDSM) layers, in which single colonies are trapped in an array, the dimension of each layer is around 2–3 cm long, 7–8 cm wide and 3 mm thick. This, in turn, allows for the preparation of high-throughput experiments using these chips, since several different conditions can be studied at once in a small space. One of these studies, for optimization, examines light cycle and light intensity variability in microalgae cultivation. Thanks to the flexibility of construction through layers, the devices can be adjusted with an additional light-blocking layer, light–dark cycle control layer or light intensity control layer [23]. The devices can also be modified to allow a specific flow of substrate into the growth chamber with the use of valves, which in turn can be used to screen lipid accumulation through different concentrations of a stress-inducing agent [50]. Substrate solutions with defined pH can be used although pH measurement within the chip is not reported.

A steady liquid flow is normally used during the illuminated cultivation of microalgae, but during initial inoculation, unequal flow conditions are used for diffusive mass transport into the cultivation chambers. During this process, air bubbles may cause a slight disruption of flow rates that have to be accounted for [51]. A polydimethylsiloxane membrane of 80–100 µm beneath the droplet allows the permeation of water so that its volume remains stable. This enables storage in the chip for up to 33 days [52].

The compact size of the chips allows experiments to take place near or directly under measurement devices such as a microscope. However, dead microalgae cells cannot be distinguished from living cells.

Mutant colonies of phototrophic cyanobacteria can also be studied using illuminated microfluidic photobioreactors. Thanks to high throughput experiments, different mutant strains can be studied under the same conditions at once, which allows researchers to find the most productive colony under the presented conditions. In these cases, the screening substances are added to the microfluidic chip through the feed flow. The waste from the chip is then captured and analyzed to determine the concentration of the product of interest [53].

According to the experimental requirements, the microfluidic layers present different custom-made designs, which may lead to difficulties in experiment replication. Some of these layers can also be made from different materials according to the needs of the experiment, i.e. photosensitive epoxy for a light intensity control layer [23]. However, molds are usually used to create layers all together, which allows for many chips to be used during these experiments.

### Shaken photobioreactors

**Fig. 1:**
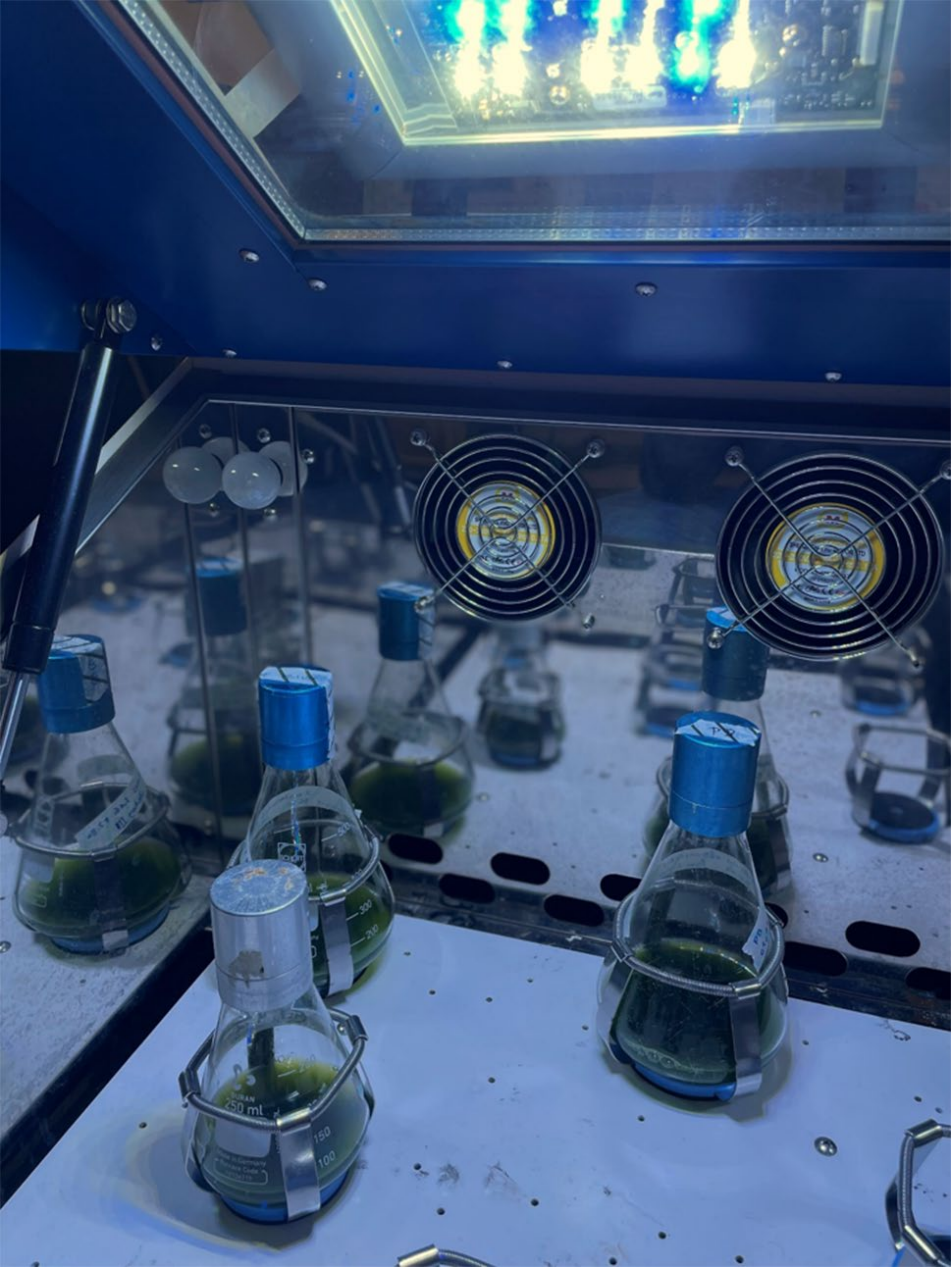
Shake flasks with 50–100 mL microalgae suspension inside a shaking incubator. A LED-panel is placed on top of the transparent incubator cover. An alternative is the individual illumination of shake flasks from the bottom up by individual LED-panels (not shown)

Two low-volume cultivation devices for photosynthetic microorganisms are illuminated microtiter plates and shake flasks. Both are standard laboratory equipment and the low volume of these cultivation devices allows for parallelized batch experiments in an incubator controlling solely the temperature. Illuminated shake flasks with microalgae are shown in Fig. 1.

Microtiter plates are available in different sizes, forms and built out of different materials [43, 54]. What they all share is their structure, which combines 12, 24, 48, or even 96 wells to a whole device. Each illuminated well operates as a single mini photobioreactor. The plate itself is placed in an incubator, for which a defined temperature and rotating velocity are set. That is why those two state variables are equal for the whole plate, while media characteristics like initial pH, nutrient concentrations, or microalgae strains can vary between single wells [55]. In the heated incubator, water evaporates out of the open wells, which is why covering has proven useful and several different covering materials exist [56]. Microtiter plates with 48 or 96 wells offer more space for differentiation of cultivation conditions and are used for screening, toxicity tests, and media optimization [54, 57]. The low cultivation volume of the single wells (< 4000 µL) results in a relatively low cost for the media components.

In microtiter plate systems, light distribution does not play a major role in microalgae growth. Light path lengths in the suspension are short and orbital shaking takes care of adequate cell distribution in the medium. The addition of beads can additionally be helpful if the cells tend towards agglomeration [58]. Recently, most publications relied on fluorescent lamps or LED illumination with light intensities of a few hundred µmol m^−2^ s^−1^ [58–60].

The major advantage of microtiter plates is their size, which allows for parallel cultivation under different conditions and easy automatization of online measurements. For most microtiter plate systems described in the literature, control of the light intensity, temperature, CO_2_ in the gas phase and mixing are possible [54, 61]. Others also allow for online biomass measurements [58, 60] or pigment ratio analysis via detecting absorption spectra [62]. One major drawback of low volume cultivation is that light attenuation is not comparable to an industrial scale photobioreactor. For this reason, microtiter plate experiments are usually followed by illuminated shake flask and small laboratory photobioreactor cultivations [43, 56]. Another drawback is that continuous sampling is not possible due to the low volume [60, 62, 63].

Illuminated shake flasks are another common laboratory-type photobioreactor. As microtiter plates, they are most often shaken on an orbital shaker, which can be in a heated incubator that provides equal temperatures for several shake flasks. Because of the mass transfer resistance of the shake flask closure and the gas–liquid mass transfer within the flask, shake flasks in general have a non-ideal gas exchange [64]. The limited availability of carbon dioxide for the phototrophic microorganisms in suspension can be counteracted to a certain degree by additional CO_2_ addition [65].

Uncontrolled shake flasks differ from large-scale photobioreactors in many characteristics, while initial medium concentrations are the same [64]. As is the case for microtiter plates, an external gas dispersion in the liquid phase is not vital for shake flask cultivation. Gas–liquid mass transfer of CO_2_ is influenced by agitation rate, liquid volume and shake flask geometry. Stronger agitation leads to better CO_2_ supply for the cultured microalgae but can damage them [66]. Enrichment of CO_2_ in the incubator atmosphere ranging from 0.04 to 1% (v/v) CO_2_ is known to contribute to higher biomass concentrations, even at light intensities, leading to decreased growth rates without CO_2_ addition [65, 67]. In general, shake flasks without baffles are suited for most applications, but those with baffles are used as well [68–71].

Illuminated shake flasks are commonly used for medium optimization [69, 72] and screening for industrially promising microalgae [34, 73, 74]. In one incubator, several different strain-medium combinations can be evaluated, while temperature and illumination variability is only possible with different incubators. As is the case for microtiter plates, illumination is accomplished with LED or fluorescent lamps that illuminate the whole shaker from above, which is the cheaper option, or from below [24, 75, 76]. In general, the biomass of photosynthetically active microalgae and cyanobacteria in shake flasks tends to be higher under higher light intensities in the range of 50 µmol m^−2^ s^−1^ to at least a few hundred µmol m^−2^ s^−1^ [24, 77]. But since light limitation conditions are preferred in industrial-scale production, phototrophic shake flask cultivations are often performed under non-ideal light intensities [30, 67]. Light intensity cannot only affect the growth of cultivated organisms, but required product composition, like lipid content, can affect it as well. Chang et al. compared the batch process performance of red microalgae *Porphyridium purpureum* in a 1 L illuminated shake flask with a 50 L aquarium tank photobioreactor. While light intensity and the temperature had a similar impact on biomass concentrations in both cases, arachidonic acid amounted to 40% of the fatty acid content in the 50 L aquarium tank photobioreactor, compared to only 20% in the shake flask [77].

Temperature control is easy to manage by adjusting the incubator temperature. But without in-line measurements, fluctuations within a few degrees Celsius are possible due to the heat produced by the illumination system. Automation of process control in shake flasks is technically possible [78], but so far it is not used for batch studies with phototrophic microorganisms. Therefore, manual sampling is the method of choice to check progression of state variables like pH, and the concentrations of dissolved oxygen, dissolved CO_2_, biomass, and products [79]. One solution for the control of varying pH within die growth media is to use self-buffering medium [54].

In the up-scaling of bioprocesses from the illuminated shaken bioreactor systems, more controlled laboratory scale photobioreactors are used with active dispersion of the gas phase inside the reactor, e.g. illuminated bubble column, flat-plate (gas lift) or stirred-tank photobioreactors [80–88].

### Bubble column photobioreactors

**Fig. 2:**
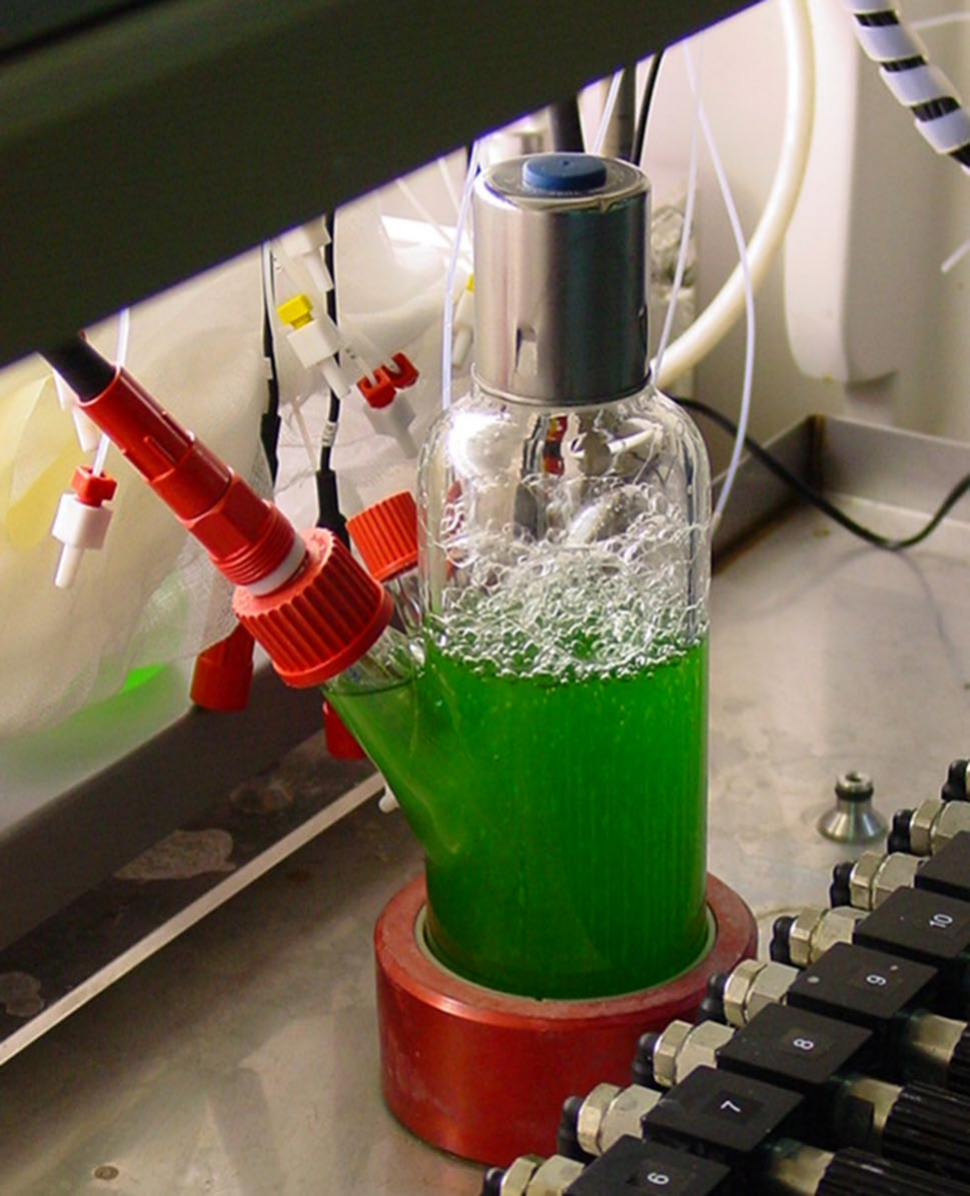
Bubble column photobioreactor with 200 mL working volume, defined gas flow and pH electrode in an illuminated incubator [109]

Usually, the bubble column photobioreactor has a cylindrical form (Fig. 2). If the bubble column photobioreactor is separated into two parts representing one with aeration and one without, these reactors are operated as gas lift or airlift photobioreactors [83, 89, 90]. While bubble column photobioreactors are commonly used in research, they can also serve as pre-culture devices [91, 92]. Usually, static spargers are applied to disperse the gas phase (air and carbon dioxide) at the bottom of the bubble column photobioreactor [93]. Many sparger variants are used, shaped like rings [94] or plates [95] with orifices, nozzles [96] or evenly spread silica air diffusers [97], which work like porous solids. To control the speed and size of the gas bubbles dispersed in the microalgae suspension, compressors and gas flow meters are combined with controllers [85, 94, 98, 99]. The gas flow rate is usually controlled between 0.1 and 1 vvm [84, 93, 100]. The air dispersed at the bottom can have an atmospheric composition [101] or sterilized air can be enriched with carbon dioxide [84, 100]. One method is to mix ambient air with pure carbon dioxide in the needed proportions controlled by a mass flow controller [85, 95].

Bubble column photobioreactors with diameters of 7–24 cm are illuminated by fluorescent tubes [83, 95, 102] or LED lamps [84, 85, 96]. Light sources are distributed around the cylindrical bioreactor to achieve a homogenous illumination [83, 93–95]. The incident light intensity decreases from the surface to the middle of the cylindrical bubble column filled with the microalgae suspension which can lead to light-limited microalgae growth in the center and photo inhibiting effects close to the surface [96]. Hence, an alternative is internal illumination of bubble column photobioreactors using evenly distributed light tubes parallel to the axis inside the reactor [103] or floating wireless light emitters suspended in the photobioreactor [104]. Heining et al. compared bubble columns with a diameter of 5 cm with external and internal illumination. Using the same relative incident light intensity per reactor volume, a more homogenous illumination of the suspension was obtained with internal illumination [104]. Incident light intensities usually applied with bubble column photobioreactors are between 15 and 220 µmol m^−2^ s^−1^ [96, 100, 102].

Bubble column photobioreactors are usually made of transparent material if external illumination is applied. Typical are plastics like polyvinyl chloride [105] or plexiglass [90, 94, 96] and glass [84, 93, 102]. Both plastics are cheap [106]. Glass is a very resistant material and is the only one that allows thermal sterilization. If the illumination is internal, the photobioreactor can be made of untransparent material due to the direct contact of the lamps to the cultivation. Therefore, a stainless steel reactor can be used [99]. Furthermore, there are different reactor sizes reported depending on which experiments were done. Common working volumes are between 1 and 10 L [83–85, 89, 100, 102, 107] but also greater volumes of up to 28 L were operated with internal illumination [97, 105, 108]. The smallest column described contains a culture volume of 450 mL [98].

Often, experiments are done with different conditions to compare the settings of parameters [83, 93]. Parallelization could be a way to examine these at once. An incubator with 16 parallel columns has been reported testing different light intensities [109], but the gas flow settings can also be individually adjusted.

Temperature is controlled by transparent double jackets around the bubble column [95] or the bubble column photobioreactor is placed in an incubator [109]. Another possibility is the use of stainless steel tubes as a heat exchanger inside the bubble column [96]. On-line measurements of temperature, pH and DO by sterilizable electrodes are used for process control [93, 107, 108]. Also, light irradiance can be measured with quantum sensors [96]. Many bubble column reactors are equipped with valves for sampling [102, 105]. Some of them can be flushed with water to ensure representative samples [105].

Gassing of bubble column photobioreactors can lead to shear stress for the microalgae cells in suspension, which may cause interferences of the cell functions or even cell death if microalgae without cell walls are cultivated. Increasing the aeration rate leads to rises in the shear rate [103]. Furthermore, the shear rate depends on the configuration of the bubble column [110, 111].

### Flat plate photobioreactors

Flat plate photobioreactors represent a common reactor type in medium-scale studies of microalgae. Made of two joined plates with a small and constant distance between them, they offer a large surface for light radiation and therefore light usage efficiency is high and dark zones can be avoided in the microalgae suspension. In different approaches, the flat plates are placed vertically, horizontally or at certain angles. Like bubble column photobioreactors, flat plates can be operated as airlift photobioreactors with separated areas, one of which is aerated [112–114].

Air is distributed via spargers [112, 115–119], tubes [113, 120] or membranes [121] for mixing and carbon dioxide supply. In some works, additional mixing tools were used. Vogel and Bergmann used static mixers [122]. Huang et al. improved the mixing performance of a photobioreactor with inclined baffles [112] and Wang et al. compared the usage of inclined baffles to horizontal baffles resulting in a better mixture and light distribution with inclined baffles [123]. With gas flow rates from 0.1 to 1 vvm, there is no difference to bubble columns [81, 86, 114, 119, 120].

Cultivations in flat plate photobioreactors with gas mixtures containing ambient [101, 117] or higher carbon dioxide concentrations [116, 121, 122, 124] have been reported. Cordara et al. mixed carbon dioxide and nitrogen with a gas mixing system [82]. The addition of CO_2_ can be used to control the pH in photobioreactors because CO_2_ consumption by the microalgae results in a pH increase (e.g. [26, 47, 91, 125–127]). Besides, the supply of carbon dioxide, humidification of the inlet gas phase can be applied to reduce evaporation [71, 120].

LED lights are commonly used for the illumination of flat panel photobioreactors [82, 86, 91, 116, 117, 120, 124, 128–130], but other light sources like OLEDs [71], sodium vapor lamps [122], fluorescents [112], halogen lamps [131] and neon lamps [113] have been used, too. The illumination of the thin reactors is mostly provided from one side [82, 91, 113, 121, 122, 128–130] but flat plate photobioreactors with two-sided lighting have been implemented as well [112, 116]. For the reflection of light, Delavari Amrei et al. operated with mirrors on the other side of the reactor instead of using a second light source [132]. Many operations in flat plate photobioreactors are done with light intensities between 80 and 400 µmol m^−2^ s^−1^ [101, 115, 118, 122].

**Fig. 3:**
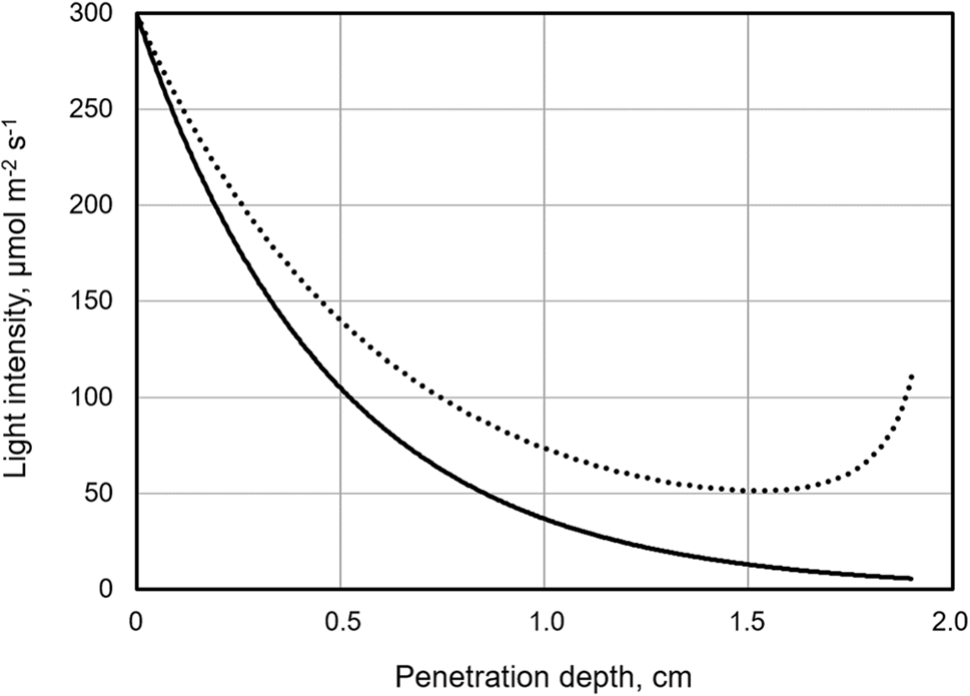
Modeling of light attenuation in the microalgae suspension. Exemplary comparison between a flat PBR (solid line) e.g. flat plate PBR [91] and a cylindrical PBR (dotted line) e.g. bubble column PBR ([83]). Light attenuation in the cylindrical reactor is influenced by the focusing effect [133]. Process parameters for both calculations were set to: incident light intensity *I*_0_ = 300 µmol m^−2^ s^−1^, optical density OD = 3.0, absorption coefficient 70 L m^−1^, layer thickness flat PBR 2 cm/radius cylindrical PBR 2 cm

Comparing light distribution in flat panel photobioreactors with cylindrical bubble column photobioreactors illuminated from outside shows that light attenuation by the microalgae in suspension is much more pronounced in flat panel reactors because light attenuation in the cylindrical bubble column reactors is compensated by the geometrical effect of light focusing towards the cylinder axis (Fig. 3). These effects are discussed in detail by Jacobi et al. [133].

Many researchers investigated the effect of light intensity on phototrophic microorganisms. The light intensities can be varied to identify the optimum for the growth and product formation of microalgae [82, 86, 91, 128, 129]. Furthermore, changes in the light intensities during the cultivation have been made [47, 114]. For scale-up, the physical dynamic simulation of day and night cycles of the sunlight should be considered. Therefore, Wolf et al. simulated day and night by the dynamic modification of the incident light between 0 and 1850 µmol m^−2^ s^−1^ to mimic a Mediterranean summer day in June [47]. Most works deployed a simplified constant illumination with 12 h daily [81, 118, 131] but light cycles with a ratio of 14 h light and 10 h dark have been reported as well [101]. Three different light/dark cycles (24 h: 0 h, 16 h: 8 h, 12 h: 12 h) have been investigated for wastewater treatment with *Chlorella vulgaris*. Both biomass productivity and removal of nitrogen and phosphate increased with higher light/dark ratios [117].

To gain wavelength spectra similar to the sun, often white LEDs are utilized. Light intensity is identified within 400–750 nm [47], from 400 to 800 nm [91] or from 400 to 700 nm [134]. But some experiments are performed only with red–orange light because of its high impact on growth [82]. In flat-panel photobioreactors, Wagner et al. investigated the effects of red, green, and blue light as well as their dichromatic combinations on the growth and photo conversion energy of *Chlamydomonas reinhardtii* [121]. It was evidenced that a combination of red and blue lights with a ratio of 90 to 10 leads to optimized biomass production at low incident light intensity but with an increase of light intensity, white light was identified as more effective.

For temperature control, transparent double jackets [101, 114] are used as heat exchangers or the flat plate photobioreactors are placed in water baths [122, 135]. Small flat plate photobioreactors can be put in temperature-controlled incubators [71, 86, 121]. Furthermore, direct electric elements have been reported, too, representing automated on- and off-turning radiating heaters [115] or Peltier elements [124, 129].

The pH, DO and temperature are often measured online with sterilizable electrodes [82, 115, 122, 128, 129]. In some flat plate photobioreactors, the measurement of the optical density is applied during the process [82, 128]. Furthermore, the chlorophyll content may be identified online via fluorescence measurements [71]. The remaining light intensities after passing the microalgae suspension in flat panel photobioreactors can be measured online with light meters [123] or spectroradiometers [91, 131]. The emission spectrum can be measured with a diode array detector [121].

Samples for off-line analytics are sucked via tubes out of the culture medium [82, 101, 122]. Other methods reported are via needles inside the plates reaching the liquid phase connected to valves and with sterile syringes [121] or seals containing a port for sampling [71].

Reactors can have sizes ranging from a few hundred milliliters to deciliters [86, 117, 122, 124, 129, 130]. Many reactors are commercially available with different volumes [81, 82, 91, 122]. Krujatz et al. described in their experiments a flat plate photobioreactor with the smallest known capacity of 15 mL so far [71]. Frames of stainless steel are normally used to hold the transparent plates [121, 124]. Disposable flat plate photobioreactors are available as well [135]. They are made of cheap polyvinyl chloride and need no metal frame.

Typical materials for the plates of flat plate photobioreactors are plastics like plexiglass [71, 101, 117, 130], polyvinyl chloride [122] or polycarbonate [115] and glass [118, 121, 124, 136]. Polycarbonate is transparent like the other materials and provides a high glassing temperature [106]. The choice of materials is also important to prevent biofilm formation. Melo et al. compared different materials in pretests resulting in a low adhesion of *C. vulgaris* on stainless steel and a high adhesion on polyvinyl chloride [137]. Comparing two flat-plate photobioreactors, one with rough and one with smooth polyvinyl chloride surfaces, resulted in a 20% higher amount of biomass in the reactor with a rough surface. Gassing of flat plate photobioreactors can lead to shear stress for the microalgae cells in suspension. High shear forces can lead to cell death if microalgae without cell walls are cultivated [138], but low shear forces lead to a lack of mixing and biofilm formation [130]. Belohlav et al. presented the biofilm formation dependence of *C. vulgaris* on the flow rate and shear stress leading to a nearly fourfold increase of biofilm by reducing the flow rate about four times and the shear stress nine times [130].

### Stirred-tank photobioreactors

**Fig. 4:**
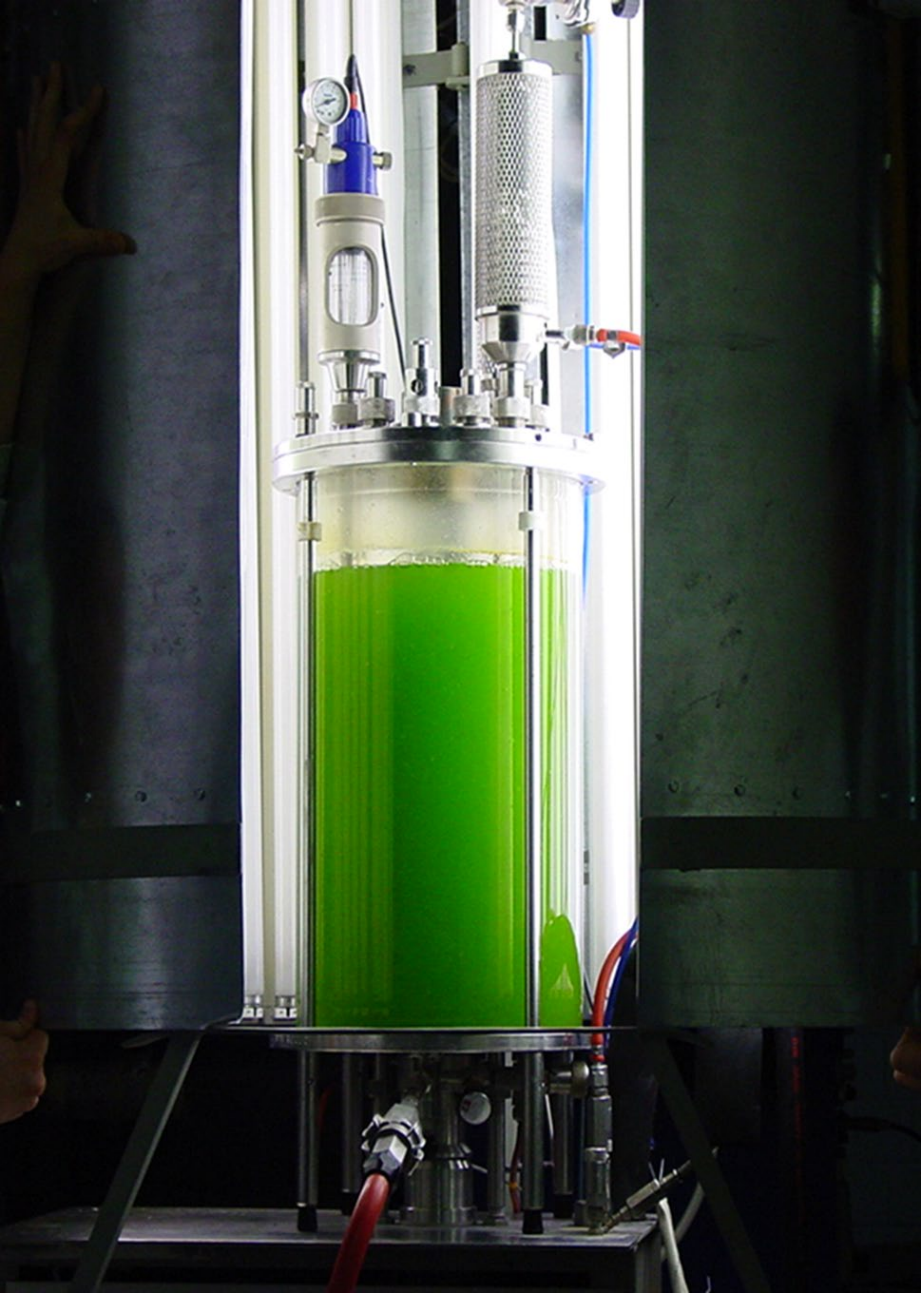
Stirred-tank photobioreactor on a liter-scale with external illumination

Standard lab-scale stirred tank bioreactors made of glass can be used to cultivate microalgae algae if an external or internal illumination device is installed in addition (Fig. 4). The diameter of the stirred-tank photobioreactor with external illumination should be small enough to ensure sufficient photon flux densities in the middle of the reactor to reduce light limitation in the microalgae suspension. These photobioreactors offer a highly controlled environment for the microalgae with perfect mixing, as well as good heat and mass transfer thanks to their broad use and optimization, with the disadvantages of high differences in the local energy dissipation (shear stress) [87]. The use of shear protecting additives for microalgae cells sensitive to shear stress, like dinoflagellate, might be required for an optimal cultivation, and have been used successfully in 2 L stirred-tank photobioreactors [139]. Another example is the red algae species *Agardhiella subulata* which, while not showing a significant change in biomass production rates even with high impeller speeds, adopted a compact spherical shape during cultivation [140].

The light source of this type of photobioreactors has been the subject of experimentation. Optical fiber has been used both to distribute solar light into an indoor stirred-tank reactor coupled with an internal artificial light source for the night and cloudy days [141]. Furthermore, optical fibers have been used as an internal light source to investigate the flashing light effects on microalgae [142]. Red light emitted by LED has also been tested in wastewater treatment with stirred tank photobioreactors, since the low energy of this light enhances photosynthetic efficiency and is barely absorbed by water molecules [143]. Due to the ample understanding and energetic optimization of stirred-tank bioreactors, online measurements of oxygen concentration at constant incident light intensity can be used in conjunction of a model to accurately estimate the biomass concentration, average light intensity in the reactor and photosynthetic efficiency. Temperature and pH are measured online and controlled in stirred-tank photo bioreactors by applying standard probes and controllers [88].

### Tubular photobioreactors

These photobioreactors usually consist of two parts interconnected by pumps: the solar receiver and the airlift system. Most of the photosynthetic reaction occurs in the solar receiver, a long tube installed in varying geometries with the diameter of tubes used in this part usually being optimized for the best sunlight capture by the microalgae suspension pumped through the tube while reducing the area needed for the tube’s installation. The airlift system, also called the bubble column, is where the excess oxygen accumulated through the metabolism of the cells is taken outside of the reactor. Usually, CO_2_ is also injected at the end of the airlift system/beginning of the solar receiver to the increase residence time of CO_2_ in the solar receiver section. A heat exchanger can also be included in this part to have better control of the cultivation temperature [144]. Laboratory scale tubular photobioreactors can also be simplified versions of this model, such as having just a solar receiver part with a simplified pumping system. This reduces costs and properly simulates a complete tubular photobioreactor [145]. The design can also be scaled up to industrial capacities easily thanks to their modular construction method [146].

The usual volume for a tubular photobioreactor on a lab scale is between 1 and 3 L and, depending on the experiment type, a fluorescent lamp might be used for the illumination of the culture. Several designs for tubular bioreactors have been developed to enable efficient light capture, the most common being serpentine bioreactors, manifold bioreactors, which connect tubes of the solar receiver at both ends to two manifolds responsible for gas exchange and distribution, and helical bioreactors which consist of small flexible tubes that surround a frame structure [147].

Vertical and horizontal tubular photobioreactors are the two main orientations of tubes used most of the time on the laboratory scale, and both can be operated continuously, semi-continuously or discontinuously. Temperature, pH and oxygen concentration can be measured in different points of the tube depending on the type of experiment, while liquid and gas flow rates are measured at the CO_2_ injection point [148].

Different challenges present themselves for this type of photobioreactor, including uniform illumination of the culture [149], low oxygen partial pressure needed for optimal growth [150], and cardinal positioning of the reactor depending on the latitude for higher light interception [151]. A tubular photobioreactor is also relatively expensive to build and sustain, particularly when it comes to mixing and cooling. One of the main challenges for the cultivation in tubular photobioreactors is the maintenance of high concentrations of CO_2_ after the injection point. The growth rate and productivity of the microalgae cells increase proportionally to the CO_2_ concentration in the solution. Furthermore, lipid concentration inside the microalgae also increases proportionally to the CO_2_ concentration, which could be important to produce biofuels through microalgae [152].

To increment the availability of CO_2_ and nutrients for microalgae cells across the tubes’ length, different methods are being considered. The addition of a porous tube (dialysis tube) in between the cultivation tube in which these substrates flow in opposite directions has been tested (tube-in-tube configuration). This, in turn, also removed excess oxygen produced by the cells [145]. Another option for higher CO_2_ concentration in the tubular photobioreactor is the use of nanofibers or other materials in the solution that absorbs more CO_2_ than water so that the CO_2_ transfer into the liquid phase across the bioreactor length can resemble the injection point. However, the nanofibers must be replaced after a couple of days to keep the optimal distribution of CO_2_ throughout the reactor, since biomass adsorption can block the CO_2_ release [153].

New designs of tubular photobioreactors have been experimented on at a lab scale, i.e. the Fibonacci-type bioreactor, which reaches a higher light utilization efficacy. This tubular photobioreactor has both a vertical and horizontal orientation of tubes and reached 1.4 increased solar radiation interception while also preserving the required pH, temperature and oxygen concentration levels for *Spirulina platensis* [154].

### Other photobioreactor designs

All photobioreactors are composed of three fundamental phases, the liquid phase in which the nutrients are delivered, the solid phase, i.e. the microalgae cells, and the gas phase for CO_2_ fixation and O_2_ removal. A light radiation field is on top of the phases and is needed for the photosynthetic process. This basic building blueprint allows for flexibility when designing new models of photobioreactors, which can be constructed according to the needs of the experiment [155].

A possible alternative for microalgae cultivation with reduced cost and inherent sterility is the use of polyethylene bags. These bags have a wide volume range and are usually hanged vertically to increase the sunlight capture. An aeration system can also be added to the bags to increase biomass yield [156]. The bags can also be placed on platforms on the ocean, which facilitates mixing by wave movement and temperature control of the culture, resulting in higher yields of microalgae biomass and intracellular products. Nevertheless, these bags usually suffer from insufficient mixing, which can lead to reduced yields at higher volumes of culture and reduced microalgae biomass and product concentration [157].

Microalgae can also be cultivated in relatively simple containers, such as aquarium tanks illuminated from the top. These containers are capable of being a somewhat improvised photobioreactor once a pump is included for improved aeration and mixing. However, it is important to note that the growth rate from microalgae in these aquarium tanks was lower in comparison to hanging bags [156].

A cheaper alternative to closed photobioreactors is open ponds operated as small-scale raceway ponds in the laboratory with paddle wheels for mixing. While these open photobioreactors are usually built with industrial capacities in mind, cultivations of microalgae in lab-scale raceway ponds illuminated from the top are most often applied to get a better understanding of growth and product formation in a down scaled photobioreactor [22].

Thin-layer cascade photobioreactors are another type of open photobioreactors illuminated from the top, where the microalgae suspension is channelled on raceways with adjustable angle. Gravitational force moves the cell suspension downwards. The suspension is collected in a retention tank and circulated by a centrifugal pump. The coordination of volume flow and angle lead to a very thin adjustable layer thickness of 0.5–1 cm. Thin-layer cascade photobioreactors are built with a single-channel or double-channel and are often used inside greenhouses [158]. A typical lab-scale thin-layer cascade photobioreactor has a surface area of 8 m^2^, which corresponds to 65 L working volume and is set to a layer thickness of approximately 0.6 cm. The design enables dynamic climate simulations with respect to light, air temperature and air humidity [159]. Complete vertical mixing can be achieved by a sufficient length of the raceway [160] or via the retention tanks, provided a relatively short cycle time is given for the specified reactor [161]. A comparable design introduced the addition of mixing rods, which would increase mixing and minimize cell sedimentation [162]. LED panels are fixed with a distance of ~ 25 cm above the microalgae suspension layer and can be combined with natural sunlight. Temperature and pH can be monitored in the suspension inside the retention tank. While pH is adjusted by mass-controlled addition of CO_2_ in the retention tank, the suspension temperature is controlled by the surroundings. Typically, DO is not measured online. Due to the large surface to volume ratio and good mixing, oxygen inhibition will not be observed in the suspension. The evaporation due to the large surface area can be automatically compensated with water. Thin-layer cascade photobioreactors are able to achieve the highest biomass densities described in the literature [159, 163].

## Scalability of photobioreactor systems

Upscaling in bioreactor systems aims to transfer the laboratory scale process performance to an industrial scale system with a higher total volume and capacity, and to specify the reactor design criteria accordingly [155, 164]. Although various types of photobioreactors, including bubble column, airlift, flat-plate, stirred–tank, tubular or other reactors have been developed so far, there is no standard design of an optimal photobioreactor for a scale-up approach.

Scaling up from a laboratory scale to a commercial unit is challenging due to the difficulty in assessing the various factors that influence the scale-up process during cultivation. Most large-scale phototrophic cultivations give a lower yield than expected from the laboratory [41, 126]. Phototrophic microorganism growth mainly requires light, inorganic carbon dioxide, water, and inorganic nutrients under suitable growth conditions, regarding pH and temperature. All of those are closely interrelated, making the design for a photobioreactor more complicated. For the scalability of photobioreactors, an appropriate setting between various factors must be carefully considered, such as light intensity, light–dark frequencies, light distribution, hydrodynamics, and environment (nutrients, pH, and temperature). Light availability for each cell might often be the most important factor for scaling up in photobioreactors [165–167].

According to Gudin and Chaumont, the geometry of the bioreactor plays a decisive role in the hydrodynamic stress, type of pump utilized, morphology of algal cells, physiological conditions of microalgae, and other properties [168]. Even though there are state variables that require standard equipment (e.g. pH) for process control, there are additionally bottleneck state variables that often cause an unsuccessful application and still limit the industrial use of photobioreactors [29, 39].

Before recommendations are made on the criteria for reactor selection and process operation during scale-up, the main problems are briefly listed in advance (Table 1). Photobioreactors have to consider light-specific factors in addition to the classical scale-up problems that also exist in heterotrophic reactor systems. In the cultivation of phototrophic microorganisms, scale-up challenges are often related to light availability in the reactor with production rates mainly dependent on it. These problems also relate to the coming recommendations and are described in more detail in the following sections. Please note that each reactor design has different challenges of varying scope.

**Table 1:** Challenges during a scale-up of photobioreactors

General scale-up problems	Specific microalgae-related challenges
Change in local reaction conditions Shear stress vs. mass transfer Contamination Biofilm formation	Homogeneous light availability Dynamics (light, temperature) Light intensity (photoinhibition/-limitation) Temperature (min./max. extrema)

### Laboratory-scale cultivation should already consider the large-scale systems in terms of reactor type, climatic, operating conditions and nutrients

Often, laboratory-scale experiments are performed with optimized strains, synthetic media, and under sterile conditions [126, 169, 170]. The upscale strategy often contains a switch from an indoor to an outdoor environment [41, 169]. This results in new problems and challenges that could not be encountered on a laboratory scale, i.e. contamination and insufficient light supply [171]. Upscaling of phototrophic processes is essentially based on surface area rather than volume, unlike heterotrophic fermentation [125, 126].

One important approach for the large-scale consideration in lab-scale experiments is to keep the ratio of surface area to volume constant, which can be achieved by maintaining geometric similarities within the scale-up [46, 126]. Flat panels and tubular photobioreactors have been established as two of the most promising lab-scale types for the transfer to a larger scale system, primarily because they have similar and large surface-volume ratios in various sizes [172–174]. The industrially established standard photobioreactors are open raceway ponds illuminated with natural sunlight [40, 175]. Open raceway ponds with a depth of 20–30 cm provide high area productivity and are therefore useful for some applications [176, 177]. Even if raceway ponds for the cultivation of microalgae typically show an insufficient mass transfer, the use of these ponds is considered more viable in large-scale systems due to their large surface, simple construction, low-cost operation, and relatively low shear stress for the microalgae cultivated [20, 126, 178]. Closed photobioreactor systems, on the other hand, have the advantage of a more controllable design of the layer thickness and show less contamination with unwanted algae and other microorganisms [179].

To keep the ratio of surface area to volume constant, process transfer to a larger scale is very often performed by increasing the number of standard units of the photobioreactor, which is also supported by Janssen et al. [180]. Their study concluded that a scale-up of tubular photobioreactors can only be done by increasing its standard units, as the reactor type is limited in size due to oxygen accumulation in the non-aerated tubes. Koller et al. used this approach of scaling up by increasing the units for flat plate reactors to keep the surface-to-volume ratio constant during scale-up. The upscale included 10 flat-plate bioreactor modules with a surface area of 2 m^2^, a depth of 22 mm, and a working volume of 30–45 L each. Their process was evaluated using process performance data from 1.8 L geometrically similar laboratory-scale LED-illuminated photobioreactors with a surface area of 0.09 m^2^ and a depth of 20 mm [126]. For the whole approach of scaling up by increasing the units (“numbering-up”), the costs of operating several smaller units must be weighed against their benefits in the individual application.

In terms of light supply, artificial lighting can be advantageous, especially for laboratory-scale experiments [27, 28]. Validation of strains and parameters in preparation for upscaling is often done under artificial light with the possibility to adjust it to a specific wavelength, intensity, or other properties [81, 181]. For artificial light source selection, light-emitting diodes (LEDs) are preferred, because they are more energy efficient than conventional lighting and provide better performance and operational control [182]. From an economic perspective, the artificial light supply in upscaling leads to additional costs. An experiment with purple phototrophic bacteria (PPB) estimated the lighting costs using artificial LEDs to be $1.9 per kg of PPB biomass (assuming a maximum empirical value of 59 g COD/kWh for biomass energy yield) [183]. This often leads to the use of sunlight during an industrial scale-up, as it is a free source of light. For testing the outdoor lighting conditions for industrial reactors without an artificial light supply, it can be advantageous to simulate the lighting conditions of the sun beforehand in the laboratory. Leonardi et al. present a methodology to reproduce the solar lighting conditions of a vertical bubble column reactor located in Santa Fe, Argentina, with a programmable LED module attached to a commercial laboratory reactor [184]. This approach can be beneficial to simulate the conditions of a scale-up supplied with sunlight. Here, the outdoor and indoor average volumetric rates of photon absorption were similar. Comparisons were made regarding the distribution and average volumetric absorption rates of PAR (photosynthetically active radiation) photons for different scenarios. Another approach for climate simulation is presented by Apel et al., for phototrophic cultivation conducted at the TUM AlgaeTec Center [159]. The Center consists of three glass halls with a pitched roof, air conditioning for the dynamic controlling of temperature and air humidity, and LED lamps. The microalgae are powered by both solar and LED radiation to save energy and use the most natural spectrum of sunlight. In contrast to other climate simulation light sources for microalgae, the TUM AlgaeTec dynamic light simulation shows an accurate spectral match over the whole PAR wavelength range. Ogbonna et al. present another example of a scalable, internally illuminated stirred tank photobioreactor. The light input coefficient—and thus, the productivity—was kept constant using a device that collects sunlight and distributes it via optical fibers in the reactor. It was equipped with a light tracking sensor so that the lenses rotate with the position of the sun. This makes it possible to use sunlight for photosynthetic cell cultivation in indoor photobioreactors [141].

One aspect to design more economically feasible microalgae processes is to use nutrients from wastewater and flue gas as CO_2_ source [185]. Effects on important state variables for growth and product formation by the microalgae caused by the wastewater should be analyzed beforehand in lab scale PBR [143]. Dual-purpose microalgae bioprocesses that produce lipids or other products while treating wastewater are more likely to become economically viable [186].

### Robust microalgae should be selected beforehand to avoid scale-up failure by contamination

As open systems, like raceway ponds, are among the most widely used large-scale photobioreactors, the use of microalgae with a competitive advantage to avoid contamination is a critical factor. Algal cultures are found in only a few applications of monocultures, whereas all mass microalgal production systems inevitably contain multiple non-target microorganisms (contaminants) [187]. Assuming that a lab-scale (100 mL) culture takes about 5–10 days to grow enough to be pre-culture in the next step [103], it takes at least 1 month to go from a 10 mL stock culture to having enough inoculum for a 10 m^3^ culture (assuming a scale-up factor of 10). Having these many steps and time required, not only adds to the cost but also increases the risk of contamination.

Microalgae that grow under extreme conditions can be applied to exclude competing organisms. Growing species with highly selective environmental requirements, such as *Dunaliella salina* (high salinity) or *Arthrospira platensis* (high alkalinity), is one option to reduce the risk of contamination [188–190]. A detailed assessment of the screening and selection of algal species and strains suitable for large-scale commercial culture can be found in [190].

However, contamination is inevitable and requires constant propagation of the seed culture, plus proper monitoring to keep the culture of choice dominant. One important recommendation is that potential microalgae strains should be tested under outdoor conditions at an early stage of the strain selection and evaluation process. It could be advantageous to cultivate different species with optimal growth at different temperatures in climates with large variations in outdoor temperature and solar radiation [191].

Another approach to avoid contamination is genetic engineering by making the microalgae more resistant against contamination [192, 193]. Even if phototrophic genetically modified organisms have a high potential [194], in most of the application markets for microalgae (i.e. food processing), genetic engineering is not widely accepted [179].

To prevent contamination, another approach concerns the interaction of different organisms, given by Fulbright et al.. Their study observed complex bacterial communities in algal cultures. As a result of the different scales of photobioreactor systems with different microalgae, the study recommends investigating research on the bacterial functions in algae cultures, as it is critical for successful large-scale algae cultivation. Bacteria that are harmful to algal growth must be identified, tracked, and minimized [169]. On the other hand, a probiotic culture supplement of bacterial communities can promote algal growth and stability [169, 195].

Alternatively, other strategies have been implemented for reducing negative process effects by contamination. Early harvesting or the use of chemical, biological and physical treatments are often applied to reduce or prevent the negative impact of contamination [20]. Applied methods are also filtration, UV, and the chemical pretreatment of the water source in the cultivation [194].

### Light-dependent microalgae growth kinetics should be the basis for model-based scale-up

One of the main challenges in upscaling is the light-shading and the reduced growth rate of phototrophs related to light limitation. Specifically, the light and energy per cell is a critical factor [49]. The concept of areal density accounting for the self-shading effect was first introduced by Soeder in 1980. High areal biomass densities imply low productivities because of significant light limitation and a high contribution of respiration and endogenous biomass consumption [196]. When biomass densities are too low, the light intensity can go above a critical value and the growth can be inhibited by the damaging effects of radiation (photoinhibition or photo-oxidation) and the light will be wasted as fluorescence first and then as heat [155, 196].

**Fig. 5:**
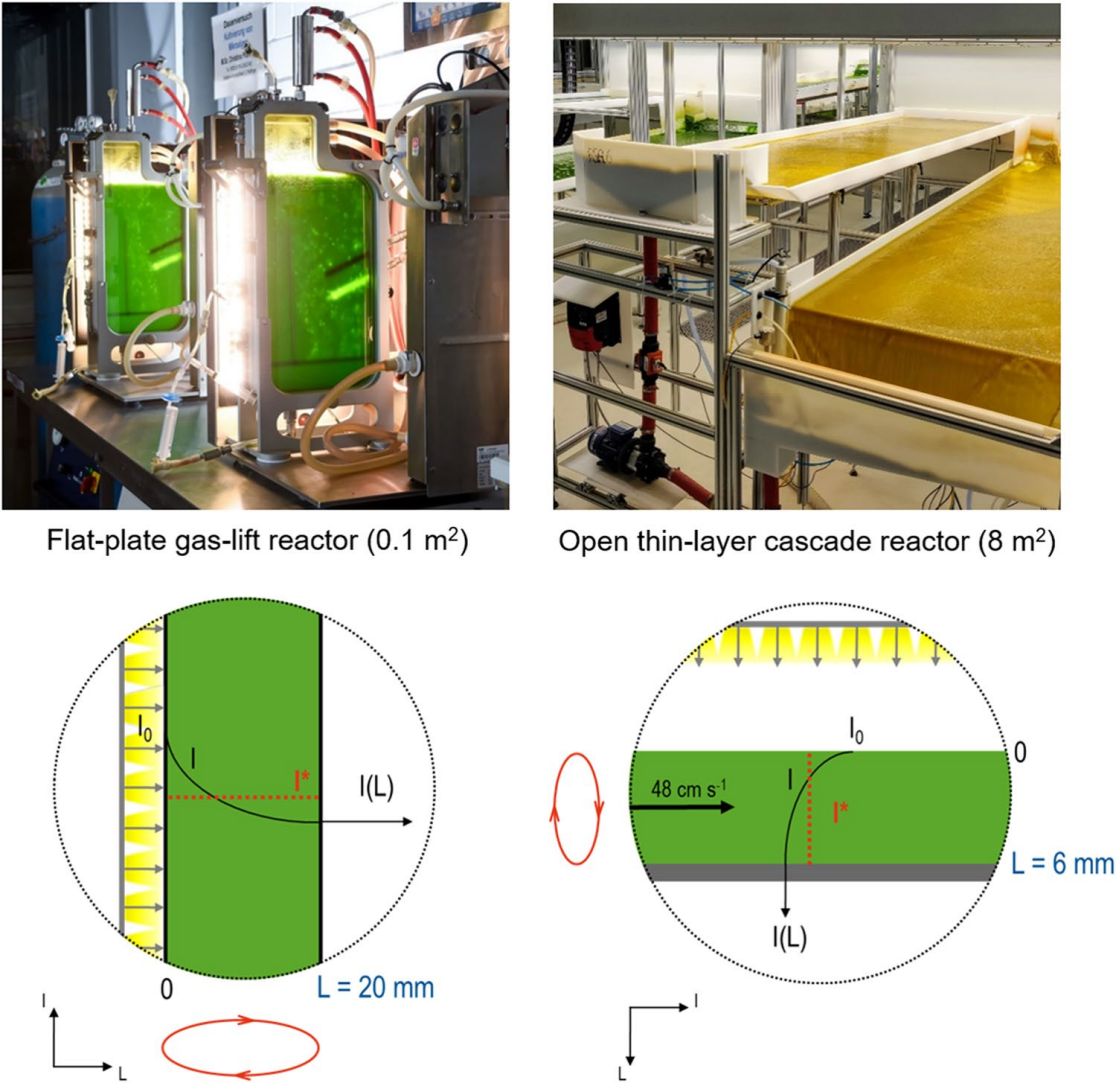
Growth kinetics identified with closed flat plate gas-lift reactors illuminated from one side (left) can be used with completely different photobioreactors like open thin-layer cascade photobioreactors illuminated from the top (right) independent of the liquid layer thickness (e.g. 20 mm with the flat plate photobioreactor, and 6 mm with the thin-layer cascade photobioreactor, respectively) if mixing is high enough (turbulent flow). *I*_0_ incident photon flux density, *I* light attenuation in the microalgae suspension, I* mean photon flux density, *L* layer thickness [25]

The kinetic data for scale-up can easily be determined in closed flat-plate gas-lift photobioreactors at known liquid layer thickness in the lab. Initially, the light absorption of the individual microalgae strain in the suspension has to be measured applying a suitable physical model, which can be Lambert's law. Assuming turbulent liquid flow, a mean photon flux density can thus be determined as a function of the algal concentration, the layer thickness of the liquid, and the light intensity at the surface of the suspension. Usually, batch processes at different constant incident light intensities are applied for estimating the growth rate of microalgae. In the exponential growth phase at constant incident light intensity, the mean photon flux densities are additionally averaged as a function of process time resulting in the mean integral photon flux density. The exponential growth rates are plotted against the mean integral photon flux densities to identify the model parameters of a kinetic approach to describe light saturation and light inhibition kinetics (Fig. 5). In addition, model parameters describing the effect of temperature on microalgae growth can be identified by performing similar batch studies at different temperatures [91, 125–127].

Growth kinetics identified with flat-plate gas-lift reactors can also be used with completely different photobioreactors if a defined liquid layer thickness can be assured, as was shown in the example of thin-layer cascade photobioreactors (Fig. 5) [26]. However, to be able to identify realistic growth kinetics, a fixed day and night cycle should be applied at the lab scale, otherwise the growth rates of microalgae are significantly underestimated. In addition, increasing the salinity as a function of process time is necessary for the closed lab-scale photobioreactors to simulate the evaporation of open photobioreactors [47]. Coupling mass balances with the kinetics of microalgae growth and product formation enables the simulation of batch and continuous phototrophic production processes with microalgae under dynamic diurnal variations of incident light and temperature in thin-layer cascade photobioreactors [197].

The optimal areal density and volumetric productivity of microalgae in suspension are influenced by the incident light intensity and the light path in the suspension (layer thickness). Degen et al. found, for example, that a reduction in the light path from 30 to 15 mm increased biomass productivity of *C. vulgaris* 2.5-fold in a flat-panel photobioreactor [198]. These results are consistent with those of Hu, et al. [172]. In a light-limited system, the shorter the light path, the higher the optimal cell density. Since a reduction of light path length from 30 to 15 mm, reduces the culture volume by half, a more than twofold increase in volumetric productivity results in an even greater increase in area productivity. In a few cases, the light does not penetrate more than a few centimeters into a dense culture of microalgal cells. There, the density of the microalgae plays a dominant factor in photosynthetic productivity. The light intensity decreases almost exponentially with the distance from the irradiated side of the reactor [199]. Lambert's law states that the loss of light intensity, as it propagates through a medium, is linear to the total intensity and path length [200]. To achieve high cell densities, the thickness of the reactor should be as small as possible [46]. In an upscale approach of outdoor raceway ponds, Grobbelaar describes euphotic depths (depth at which 1% of surface irradiance is present) which exceed the cultivation depth, result in wasted light energy and thus lower productivity. To achieve the best productivity, they recommended concentrations in the raceway ponds with *Spirulina* areal densities of about 75 g cell dry weight m^−2^ [41]. Grobbelaar et al. used a model to describe the production of green algae and showed a maximal productivity of *Scenedesmus obliquus* and *Coelastrum sphaericum* at an areal density of 38–41 g m^−2^ [201]. This implies that achieving the best productivity requires tight control of areal biomass densities over a narrow optimal interval that should be determined for each species, strain, site, and season**.**

### The scale-up factor should be adjusted to the possible level of photoinhibition during cell expansion

To minimize photoinhibition immediately after inoculation, the scale-up factor should be carefully selected. A factor of 10 was recommended as a successful transfer factor to go from a lab-scale to the next larger scale photobioreactor. Giving an example, the process can be made from shaken flasks with 100 mL to medium size reactors (1–10 L), followed by a transfer to photobioreactors on a pilot-scale (10–100 L) and an upscale to an industrial cultivation system up to 1 m^3^ [48, 132, 202]. For some very light-sensitive species such as strains of the cyanobacteria, the scale-up factor might be reduced to minimize photoinhibition after inoculation [48]. In some papers, the factor is further reduced [22] or increased [103, 170], depending on the exact process parameters, as the factor must be adjusted to the system.

### Active temperature control should be avoided by operating conditions and the selection of robust microalgae

A critical factor is a fast-rising temperature in thin microalgae suspensions due to the illumination especially in non-controlled outdoor photobioreactors [155, 202]**.** The increase in temperature influences the metabolism and growth of the microalgae and thus has an impact on their overall productivity [48]. In outdoor systems without any cooling concepts, temperatures reach a level that may be 10–30 °C higher than the ambient temperature in the summer [20]. Water evaporation in open photobioreactors will reduce temperature increase at high solar irradiation with decreasing layer thickness of the microalgae suspension, making thin-layer cascade photobioreactors advantageous compared to raceway ponds (1–3 °C higher than the ambient temperature in the summer) [203]. The optimal temperature range for the growth and product formation of microalgae depends on their type. However, robust types can tolerate a water temperature between 16 and 35 °C [47, 204–206]. For large open cultivation volumes, many authors consider temperature control not economically feasible [190, 199, 207]. Here, suitable approaches for temperature challenges and control need to be tested and adapted to the laboratory-scale reactor.

Even production processes for high-value products like ß-carotene, lutein, astaxanthin and others need to be very cost-efficient to be economically feasible. Therefore, open pond photo bioreactors are often preferred [208]. In a large-scale production of astaxanthin with *Haematococcus* spec., temperature control was applied by spraying cold water directly onto the tubular photo bioreactor to keep the temperature of the microalgae suspension below 32 °C [209]. One approach to mitigate day-night temperature fluctuations in open-pond photobioreactors in a cost effective way is to pump the microalgae suspension into a 1.5 m deep canal at night to reduce heat transfer to the surrounding [210].

One approach for the adaptation of microalgae cultivation processes to annual environmental temperature variations is to cultivate a “summer strain” and a “winter strain” [211]. It is highly recommended to select a strain that can grow well under the temperature conditions prevailing in the culture system at the production facility site, as the temperature cannot be controlled in large flow-through ponds [48]. In temperate regions, cultivation systems can also be placed in greenhouses to affect the environment around the reactor [159]. Although these approaches seem efficient, they can increase the capital and operating costs and negatively impact the environmental footprint through excessive energy and water use. Shading to maintain the temperature of a whole photobioreactor needs to be weighed up against a decrease in light utilization.

A relatively new approach investigated the integration of photobioreactor technology in building facades. This integration offers various benefits in terms of thermal management of both reactors and buildings. Energy exchanges between the building and the reactor itself can be designed to cool or warm each subsystem [212].

### Flashing light effects should be considered if positive effects on microalgae process performance has been observed

Flashing light effects have been shown to improve the efficiency of algal cultures under certain conditions by supporting the dynamic process of photosynthesis and lead to an increase in biomass formation. Several investigations have been done to understand the effect of fluctuating light on cell physiology [213–216]. In 1932, Emerson and Arnold first found that microalgae cells can exhibit the same maximum rates of oxygen production and carbon dioxide uptake under a sequence of short light flashes as under continuous light [217]. However, the frequency of the flashing light between light to dark was later shown to be a decisive factor. If the frequency is not optimized, a decrease in biomass formation can occur [218]. It was also shown that the biomass yield and growth rate were strongly affected by the light fraction (amount of the time the microalgae cells are exposed to the light divided by the total flashing light time) [176]. Therefore, the combination of different frequencies (10–100 Hz) and light fractions (0.1–1) [176], respectively 1, 5 and 10 Hz and light fractions of 0.1 and 0.33 have been studied [218].

Besides flashing light illumination by LEDs, an indirect periodic flashing light can be induced by the hydrodynamic transport of microalgae in photobioreactors [199]. Thus, algal cells are induced to cycle repeatedly between the well-lit surface or peripheral area and the dimly lit inner area of the photobioreactor. Especially photoinhibition under high irradiance can be prevented [181, 198]. Degen et al. compared microalgae growth in two flat-panel photobioreactors with identical volume and shape and single-sided illumination. One has alternating horizontal baffles to the front and to the back, which lead to circulating fluid and therefore to a defined mixing pattern within the subdivided chambers. This setup effectively simulated a regular flashing light. The reactor without baffle results in the chaotic light–dark movement for the microalgae. The volumetric biomass productivity was shown to be about 1.7-fold higher in the flat panel with horizontal baffles [198]. Abu-Ghosh et al. combined the flashing light with continuous lighting in an experiment with *D. salina* and demonstrated a successful method that can theoretically be applied in large-scale cultivation [219]. Nevertheless, the occurrence of a flashing light effect has not been unequivocally proven yet in the pilot or production scale [41, 180, 181].

### An optimum balance between shear stress and mixing should be identified

Mixing in photobioreactor systems guarantees that all cells are equally exposed to the light and promote gas–liquid mass transfer. It is important that the technology implemented on the lab scale for mixing results in a similar volumetric power input or maximum local energy dissipation rate compared to the large scale. Other than in heterotrophic cultivations, excessive mixing may damage the cells and result in culture collapse if the microalgae are susceptible to the shear force [220].

Finding a balance where mixing and aeration are sufficient for optimal microalgae growth without compromising cell integrity is essential. To reduce mechanical mixing, aeration is used on one hand for lowering the shear stress during mixing [80, 103], and on the other hand for providing gas–liquid mass transfer during the cultivation of phototrophs. The aeration rate needs to be optimized for maintaining an optimum dissolved carbon dioxide (DCD) and dissolved oxygen (DO) balance. Ding et al. reported a volumetric air flow rate of 0.2 vvm as an optimal rate for *C. vulgaris* and 0.3 vvm for *Chlorella protothecoides* in a 50 L cylindrical air-lift photobioreactor [103]. If a bubble column photobioreactor is used, Khoo et al. concluded that the combination of hydrodynamic effects and mass transfer played a significant role in the cultivation. They recommended an optimal compressed air aeration rate of 0.16 vvm suitable for the microalgae *C. vulgaris* [221].

Shear stress can also be induced by the pump components in the system [80]. Mixing by pumping is efficient but can also cause high shear forces due to fluid circulation, micro-vortexing, and mechanical stress. Mechanical stress is particularly damaging within the pump itself, with shear stress due to friction between cells and the pump walls and internal surface (i.e. impeller surfaces of a centrifugal pump) [222, 223]. Wang and Lan compared a centrifugal pump and an airlift pump in a tubular photobioreactor in which the culture is agitated with air. Here, the stress on the cells is induced by the shear association with the fluid flow and the stress when the air bubbles burst at the fluid surface [80]. It was shown that laboratory-scale cultures of *C. vulgaris* and *Scenedesmus dimorphus* circulated with an airlift pump, had higher effective growth rates than with a centrifugal pump. For both *C. vulgaris* and *S. dimorphus,* the centrifugal pump caused a reduction in cell growth, while the airlift pump had a negligible effect [224]. On the other hand, there are also contradictory study results, showing that airlift pumps are much less efficient at mixing than centrifugal pumps [225].

To reduce the damaging shear stress, an option is to choose a less shear sensitive microalgae, as shear sensitivity varies between microalgae species and depends on several factors such as cell size, presence of flagella morphology, and the presence and composition of the cell wall [80]. Wang and Lan compared the shear tolerance of different publications, showing that shear tolerance is greatest in green algae, followed by cyanobacteria, haptophytes, red algae, and diatoms. Dinoflagellates are the most sensitive species [80].

### The wall shear stress should be aligned to prevent biofilm formation

Koller et al. described that during an upscale of a batch process from laboratory scale flat-panel gas-lift reactors (1.8 L, 0.09 m^2^) with *Scenedesmus* strains to the geometrically equivalent pilot scale (300 L, 14 m^2^), a biofilm formation was observed solely at the pilot scale [126]. Biofilms most likely affected the reduction in biomass concentration in suspension at the pilot scale compared to the lab scale [159].

To prevent the biofilm formation beforehand, sufficient wall shear stress disrupts the stability of the biofilm or reduces its formation by a considerable amount. Belohlav et al. could reduce the amount of microalgae cells (*C. vulgaris*) fixed in the biofilm from 70 to 19% by increasing the flow rate of the microalgae suspension by 71% (from 45 to 77 L min^−1^) in a tubular photobioreactor. Here, the microalgae formed a thin layer of biofilm on the transparent walls, which reduces the irradiation of the microalgae in suspension. The shear force on the wall to reduce the biofilm must be balanced against the critical value of the shear force for the respective microalgae. Up to a wall shear stress of 6 Pa, the stability of the biofilm was not disturbed. Lower values of critical wall shear stress are sufficient to prevent the formation of a biofilm. However, if the biofilm has already formed in the cultivation system, the value to disturb the biofilm stability must reach several tens of Pa. Therefore, avoiding the biofilm at an early stage is more beneficial, as the degradation is associated with high shear stress for the microalgae [130].

## Conclusion

Ideally, the laboratory photobioreactor would be able to exactly simulate the cultivation conditions of the projected industrial-scale photobioreactor. Thus, it always depends on the geometry of the larger scale reactor, which of the various laboratory-scale reactors is adequate to achieve useful results. Cost and the specific parameter's requirements for the cultivation of certain microalgae must also be considered when choosing lab-scale photobioreactors.

Illuminated microtiter plates are an ideal cultivation system for high throughput batch experiments at low cell densities if operated in an incubator with CO_2_ controlled atmosphere. They combine low material and application cost with the best possibilities for parallelization and automation of measurements if no pH control is necessary. Microalgae productivity and growth behavior can be like other lab-scale photobioreactors to a certain degree, but sometimes they also differ significantly.

Illuminated shake flasks offer easy handling, low-cost batch cultivation in incubators with a controlled CO_2_ atmosphere and in comparison, to microtiter plates, their volume enables intermittent manual sampling over a few days of cultivation and for first investigations of product concentrations. Even though automation of measurements is possible, it is usually not applied as it is for microtiter plates. Meaningfully operated shake flasks may show growth and product formation comparable to other lab-scale photobioreactors to a certain degree.

When experimenting with greater volumes in the lab, different kinds of closed photobioreactors are available, each with its advantages and disadvantages, but all of them can be operated in batch and continuous mode and the state variables (e.g. gassing, pH, *T*, …) can be controlled online. Illuminated bubble columns provide a soft and homogeneous mixing, with good aeration. Due to their simple design, they are an often-used photobioreactor in the laboratory. Scale-up into industrial sizes is challenging due to the light distribution caused by the radial geometry. If the lighting is from the outside, no light reaches the center on an industrial scale, and the internal lighting does not work to use sunlight. Numbering-up of small columns instead of geometrically similar scale-up will be the remaining but costly option.

Illuminated flat plate photobioreactors are easy to build and cheap devices with simple geometry. Due to their large surface area and a short light path through the reactor, the light supply to the microalgae suspension is one of the most effective and defined so far. Furthermore, full control of state variables can be achieved with standard lab equipment. Scalability is simple by increasing the size to an economically viable ‘standard’ configuration keeping the surface-to-volume ratio constant followed by numbering-up. But due to their biofilm formation tendency, shear forces must be raised usually during operation, or special installations have to be applied.

Illuminated tubular photobioreactors are specifically built to increase the surface-to-volume ratio of the reactors, which increases the available light for the cells. These reactors can also be simplified to either simulate the solar receiver or the airlift system when experimenting on a laboratory scale to greatly reduce costs. However, uniform light capture, CO_2_ availability, O_2_ enrichment and nutrient distribution across the length of the tube are challenges when applying these photobioreactors on a large scale. Due to their biofilm formation tendency, shear forces must be raised as well, or special installations have to be applied.

Illuminated stirred tank bioreactors are well-studied equipment in bioprocess engineering and therefore easy to set up for studies with microalgae under controlled reaction conditions in the lab. However, they expose the microalgae to high local energy dissipation rates at the stirrers and some microalgae species may show no growth in illuminated stirred-tank photobioreactors due to shear stress. Scale-up into industrial sizes is not feasible because a constant surface-to-volume ratio can only be realized by numbering up.

Some other niche photobioreactor designs, such as illuminated and aerated plastic bags or aquarium tanks, are cheaper and may be interesting alternatives to traditional photobioreactor designs but more experimentation might be required and proper control of state variables may be more challenging. Numbering-up may be an option for scale-up due to the simple design.

The upscaling of photobioreactors involves challenges that also exist for conventional bioreactors. A key difference is the specific light-driven issues such as light availability, self-shading, photoinhibition, or light-emitted heat. This makes a model-based approach combining light-dependent microalgae growth kinetics with the physics of light transport in suspensions and fluid dynamics most promising for the scale-up of phototrophic production processes with microalgae. Therefore, the type of lab-scale photobioreactor used for the identification of the light-dependent microalgae growth and product formation kinetics can be different from the projected industrial-scale photobioreactor.

## Data Availability

Not applicable.
